# Prevalence and associated factors of dyslipidemia in elderly people: Results from the Ardakan Cohort Study on Ageing, Iran

**DOI:** 10.1371/journal.pone.0306388

**Published:** 2024-10-23

**Authors:** Ahmad Delbari, Shadi Naderyan Feli, Ali Reza Amirabadizadeh, Mahtab Niroomand, Mohammad Bidkhori

**Affiliations:** 1 Iranian Research Center on Aging, University of Social Welfare and Rehabilitation Sciences, Tehran, Iran; 2 Department of Epidemiology and Biostatistics, School of Public Health, Tehran University of Medical Sciences, Tehran, Iran/Iranian Research Center on Aging, University of Social Welfare and Rehabilitation Sciences, Tehran, Iran; 3 Department of Internal Medicine, School of Medicine, Shahid Beheshti University of Medical Sciences, Tehran, Iran; Mexican Social Security Institute, MEXICO

## Abstract

**Background and objective:**

Dyslipidemia is a major modifiable factor in elderly people. The study objective was to assess the prevalence and associated risk factors of dyslipidemia among the Iranian population aged over 50.

**Methods:**

This population-based cross-sectional study is part of the Iranian Longitudinal Study on Ageing conducted in Ardakan, Iran. In total, 5,197 participants aged over 50 years old were included through a stratified random sampling method. Dyslipidemia was defined in compliance with the Adult Treatment Panel III criteria. The Chi-square and independent sample t-test were used to compare categorical and quantitative variables, respectively. A logistic regression analysis was applied to determine associated factors of dyslipidemia.

**Results:**

The mean age of the participants was 62.24±7.52. The prevalence of dyslipidemia was 68.85%. High levels of total cholesterol, triglyceride, low-density lipoprotein, and low level of high-density lipoprotein were seen among 9.74%, 24.66%, 5.54%, 19.20% of the participants, respectively. In addition, 66.05% of the study participants were under the treatment of lipid-lowering medications. Among the possible investigated risk factors of dyslipidemia, male gender (odds ratio (OR) = 0.56, 95% confidence interval (CI): 0.47, 0.68), waist circumference (OR = 1.03, 95%CI: 1.02, 1.04), diabetes mellitus (OR = 2.28, 95%CI: 1.96, 2.66), and hypertension (OR = 1.59, 95%CI: 1.38, 1.83) showed a statistically significant association (p<0.05).

**Conclusion:**

Dyslipidemia is highly prevalent in Ardakanˈs older population. So, lipid management interventions are necessary for this population.

## Introduction

Dyslipidemia is one of the main modifiable risk factors for developing cardiovascular diseases [1], cancer types [2], non-alcoholic fatty liver disease [3], and diabetes mellitus (DM) [4]. In fact, dyslipidemia is a lipid metabolism disorder concerning abnormal amounts of blood lipids [5], that clinically includes one or a combination of high total cholesterol (TC), triglyceride (TG), low-density lipoprotein cholesterol (LDL-C), and reduced high-density lipoprotein cholesterol (HDL-C) [6]. Smoking, general or central obesity, sedentary behavior, hypertension (HTN), and DM are considered to be risk factors for dyslipidemia [7, 8]. The prevalence of dyslipidemia is increasing in most countries, including Iran [9]. According to the estimate of the World Health Organization (WHO) in 2018, the age-standardized mean of TC was 4.5 and 4.4 mmol/l worldwide and in Iran, respectively [10].

Aging is relevant to changes in body composition which consequently lead to considerable changes in physical fitness and health. As a result, the development and progression of diseases such as DM, HTN, atherosclerotic cardiovascular diseases, and dyslipidemia are expected [11]. Dyslipidemia existed in 33.5% of people greater than 45-years-old in a large survey among 6,038 residents of Shanghai between June 2009 and December 2012 [12]. Whereas, findings of Tehran lipid and glucose study (TLGS) showed higher levels of TC, LDL-C and TG and slightly lower HDL-C in Tehranian adults aged 20–64 years than other studies in the industrialized countries [13]. Moreover, in a study conducted from 2013 to 2014 among 3,000 men and women aged over 60 residing in Bushehr, Iran, dyslipidemia was observed among 87.8% of women and 80.2% of men [14]. Furthermore, a study carried out in 2004 in Yazd, Iran, found that the prevalence of low HDL-C, high LDL-C, high TC, and high TG was 23.9%, 11.2%, 78.8%, and 52.3% among people aged 65 to 74 [15].

According to estimates of the WHO, from 2000 to 2050, the world’s population of individuals over 60 years old will reach two billion people. Most of this increase will occur in underdeveloped countries, where the number of elderly people will increase from 400 million in 2000 to 1.7 billion by 2050 [16]. The age distribution in Iran is also changing and shortly there will be an elderly population [17, 18]. Therefore, the prevalence of age-associated conditions such as HTN, dyslipidemia, obesity, and inactivity will increase [17, 19]. In addition to change in age distribution, there have been changes in lifestyle behaviors during the last two decades in Yazd province, Iran, which lead to increase in the prevalence of non-communicable diseases [20]. In the Yazd Healthy Heart Project, conducted among 2000 Yazd citizens aged 20 to 70, the prevalence of dyslipidemia, HTN, DM, and smoking was 58.5%, 25.6%, 11%, and 13.12% respectively [15].

Awareness of the prevalence of dyslipidemia is an important stage towards improve our understanding of the burden of the problem and planning to prevent its negative clinical consequences. Considering the high prevalence of dyslipidemia in Iran and changing in the age pyramid of the population, this study was designed with the aim of investigating the prevalence of blood lipid disorders and its risk factors in the population over 50 years of age in Ardakan city, Yazd province, Iran.

## Methods

### Study design and setting

The Iranian Longitudinal Study on Ageing (IRLSA) is part of the Prospective Epidemiological Research Studies in Iran (PERSIAN) cohort [17, 21]. The current cross-sectional study, derived from IRLSA was conducted between January 1, 2020 and January 1, 2022 included people over 50 years old in Ardakan, central Iran. Participants were selected using a multistage stratified random sampling method. Initially, a list was compiled consisting of all men and women over the age of 50 who had active documents in all seven healthcare centers. Each healthcare center was treated as a separate stratum. Based on the proportional share of each center in the overall number of covered individuals, 5,197 adults aged ≥ 50 years were selected using a random sampling process. The inclusion criteria were defined as follows: over 50 years of age, Iranian citizens, a resident of Ardakan city, and favorable conditions for communicating with the research team. Moreover, participants with the following conditions were excluded: less than two years of residence in Ardakan city, people with temporary employment status, immigrants, dementia or severe mental illness at enrollment time, and living in retirement homes. Written informed consent was taken from all participants. The protocol of the current study was approved by the Ethical Committee of the University of Social Welfare and Rehabilitation Sciences (Ethics code: IR.USWR.REC.1394.490).

### Measurement of study variables

Participants were asked to provide information on their marital status and education. Age was obtained using the calendar date recorded on the birth certificate. Current smoking status (yes/no) was determined by the self-reporting of participants. Current drug consumption and duration of diabetes were also defined by the self-reporting of participants.

Weight was assessed by a fixed calibrated scale (seca 755, measurement accuracy of 0.5 Kg) with light clothes and without shoes. Height was also measured at a standing position and without shoes by a fixed measuring tape (seca 206, measurement accuracy of 1mm) in participants who are able to stand unassisted. The position of the heels, the buttocks, shoulder blades, and the back of the head were in contact with the wall. In people who were disabled in standing, ulna length, knee height, or demi-span were used as alternative measurements of height. In addition, body mass index (BMI) was determined by the following formula: weight (Kg)/height^2^ (m). Waist circumference (WC) was measured in the region between the last rib and the iliac crest by an inelastic tape with an approximation of 0.1 cm at a standing position while breathing normally.

Blood pressure (BP) was measured twice 10 minutes apart, using a sphygmomanometer (Omron M6 Comfort, measurement accuracy of 3mmHg) in a sitting position on both arms. Moreover, at least half an hour before BP measurement vigorous physical activity, drinking coffee or tea, and smoking were unallowed.

Venous blood samples were taken after 8 to 12 hours of fasting. In order to serum extraction, blood samples were centrifuged at room temperature for 10 to 15 minutes at 3000 rpm. Blood glucose and lipids were analyzed using an AutoAnalyzer. Pars Azmun kit (Pars Azmun Co. Iran) was used to measure the fasting plasma glucose (FPG). TC was measured using a Sarantashkhis kit (Sarantashkhis Co. Iran). TG was measured by a Dialab kit (made in Austria, importing company in Iran: Farsa Med Parsiyan Co.) LDL-C and HDL-C were measured by a Biorex kit (biorex fars Co. Iran).

### Diagnostic criteria

In compliance with the third report of the national cholesterol education program (ATP III), dyslipidemia was defined as TC greater than or equal to 240 mg/dL, and/or LDL-C of 160 mg/dL or more, and/or HDL-C of less than 40 mg/dL, and/or TG of 200 mg/dL or higher, and/or taking lipid-lowering drugs [22]. Classification of blood lipids levels was also done in accordance with the ATP III as followed: TC of less than 200 mg/dL is desirable, from 200 to 239 mg/dL is borderline high, and 240 mg/dL or higher is high. Furthermore, TG of less than 150 mg/dL is normal, in the range of 150 to 199 mg/dL considered to be borderline high, in the range of 200 to 499 mg/dL is considered high, and 500 mg/dL or higher is very high. LDL-C less than 100 mg/dL is optimal, in the range of 100 to 129 mg/dL is near optimal, from 130 to 159 mg/dL is borderline high, a range from 160 to 189 mg/dL is considered as high, and equal to or over 190 mg/dL considered as very high. Moreover, HDL-C of 40 mg/dL or higher is high, while less than 40 mg/dL is low.

Diabetes was identified as FPG of 126 mg/dL or higher using the guideline provided by the American Diabetes Association and/or having a definite medical history and/or being under treatment with glucose-lowering drugs [23]. HTN was defined as systolic blood pressure exceeding or equal to 140 mmHg and/or diastolic blood pressure greater than or equal to 90 mmHg and/or having a definite medical history and/or being under treatment with antihypertensive drugs [24].

### Statistical analysis

Mean ± standard deviation and frequency (percentage) were applied to describe quantitative and categorical variables, respectively. The Chi-square test was used to compare categorical variables. An independent sample t-test was also applied to compare the quantitative variables. Moreover, a logistic regression analysis was used to determine the factors associated with dyslipidemia. The variables included in the multivariable regression model were selected using a univariate screening. A univariate criterion of p-value<0.2 was considered as the stopping rule. The effect size results from logistic regression analysis were reported as odds ratio (OR) and the corresponding 95% confidence interval. All statistical analyses were performed with the STATA_17_ statistical software package. P-values of less than 0.05 was considered to be statistically significant.

## Results

A total of 5,197 participants including 2,502 (48%) male and 2,695 (52%) female, aged 50 to 99 years, were investigated in the present study. The mean age of males was significantly higher than that of females (p<0.001). Moreover, there was a statistically significant difference in terms of level of education and marital status between males and females (p<0.001) (Table 1).

**Table 1:** Demographic and clinical characteristics of the participants by gender, mean ± standard deviation or n (%).

Characteristics	Total	Male	Female	p-value*
**Age (years)**	62.24 ± 7.52	63.37 ± 7.90	61.19 ± 7.46	<0.001
**Level of education**				
Primary school or lower	3211 (61.98)	1103 (44.23)	2108 (78.45)	<0.001
Middle or high school	1378 (26.60)	909 (36.45)	469 (17.45)	
Academic degree	592 (11.43)	482 (19.33)	110 (4.09)	
**Marital status**				
Single	471 (9.06)	33 (1.32)	438 (16.25)	<0.001
Married	4726 (90.94)	2469 (98.68)	2257 (83.75)	
Body mass index (kg/m^2^)	28.60 ± 4.94	27.06 ± 4.33	29.98 ± 5.04	<0.001
**Waist circumference (cm)**	100.10 ± 11.29	99.66 ± 11.92	100.52 ± 10.66	0.008
**Type 2 diabetes**	1930 (37.25)	843 (33.80)	1087 (40.45)	<0.001
**Current using of glucose lowering drugs**	1586 (32.50)	656 (27.90)	930 (36.70)	<0.001
**Duration of diabetes mellitus (years)**	9.58 ± 24.70	10.64 ± 7.95	8.84 ± 31.53	0.151
**Hypertension**	2540 (51.55)	1077 (45.46)	1463 (57.19)	<0.001
**Current using of antihypertensives**	2288 (46.50)	939 (39.70)	1349 (52.80)	<0.001
Current cigarette smoking	1216 (24.41)	1209 (50.52)	7 (0.27)	<0.001

*Independent sample t-test or Chi-square test, as appropriate.

As shown in Table 1, BMI and WC in women were significantly higher than in men (p<0.001). Furthermore, DM and HTN was more prevalent in female participants than males (p<0.001). Instead, cigarette smoking was more frequent in men (p<0.001). The consumption of glucose and blood pressure lowering drugs was significantly higher in women than in men (p<0.001). There was no statistically significant difference in the mean duration of diabetes between males and females (p = 0.151).

Dyslipidemia was found in 3,528 (68.85%) of the participants. The prevalence of dyslipidemia components by gender is presented in Table 2. According to Table 2, a statistically significant difference was observed between males and females in terms of the level of TC, TG, LDL-C, HDL-C, and use of lipid lowering drugs (p<0.001). So that, elevated TC, elevated TG, elevated level of LDL-C, and lipid-lowering drug consumption were more prevalent among female participants. On the contrary, low HDL-C was more prevalent in men.

**Table 2:** Prevalence of dyslipidemia components by gender, n (%).

	Total	Male	Female	p-value*
**Total cholesterol**				
Desirable	3342 (66.97)	1749 (72.60)	1593 (61.72)	<0.001
Borderline high	1162 (23.29)	490 (20.34)	672 (26.04)	
High	486 (9.74)	170 (7.06)	316 (12.24)	
**Triglyceride**				
Normal	2612 (52.33)	1343 (55.75)	1269 (49.15)	<0.001
Borderline high	1099 (22.02)	480 (19.93)	619 (23.97)	
High	1231 (24.66)	554 (23.00)	677 (26.22)	
Very high	49 (0.98)	32 (1.33)	17 (0.66)	
**LDL-C**				
Optimal	1861 (41.73)	970 (45.10)	891 (38.59)	<0.001
Near /above optimal	1493 (33.48)	711 (33.05)	782 (33.87)	
Borderline high	811 (18.18)	357 (16.60)	454 (19.66)	
High	247 (5.54)	97 (4.51)	150 (6.50)	
Very high	48 (1.08)	16 (0.74)	32 (1.39)	
**Low HDL-C**				
Yes	856 (19.20)	619 (28.78)	237 (10.27)	<0.001
No	3603 (80.80)	1532 (71.22)	2071 (89.73)	
**Current using of lipid lowering drugs**				
Yes	1796 (66.05)	612 (59.53)	1184 (70.02)	<0.001
No	923 (33.95)	416 (40.47)	507 (29.98)	

* Chi-square test

As reported in Table 3, dyslipidemia was associated with gender, education, marital status, BMI, WC, DM, HTN, and smoking (p<0.05). However, there was no statistically significant difference in the mean of age and diabetes duration between participants with and without dyslipidemia (p>0.05). Further, logistic regression analysis showed that by controlling the effect of other covariates, there was a statistically significant association with dyslipidemia only for gender, WC, DM, and HTN (Table 4). So that, based on the multivariable regression model, the odds of developing dyslipidemia in men was 44% lower than in women (adjusted OR for male gender = 0.56, 95%CI: 0.47 to 0.68). With each centimeter increase in WC, the odds of developing dyslipidemia showed a significant increase of 3% (adjusted OR = 1.03, 95%CI: 1.02 to 1.04). Moreover, the odds of dyslipidemia in diabetic patients was 2.28 times that of non-diabetics (adjusted OR = 2.28, 95%CI: 1.96 to 2.66). Patients with HTN also had 1.59 times the odds compared to normotensive individuals (adjusted OR = 1.59, 95%CI: 1.38 to 1.83).

**Table 3:** Factors associated with dyslipidemia, mean ± standard deviation or n (%).

Variables	Dyslipidemia		p-value*
	Yes	No	
**Age (years)**	62.33 ± 7.58	62.08 ± 8.00	0.285
**Gender**			
Male	1507 (62.17)	917 (37.83)	<0.001
Female	1933 (74.0)	679 (26.0)	
**Level of education**			
Primary school or lower	2170 (69.62)	974 (30.38)	0.009
Middle or high school	895 (67.14)	438 (32.86)	
Academic degree	365 (63.48)	210 (36.52)	
**Marital status**			
Single	355 (77.17)	105 (22.83)	<0.001
Married	3085 (67.42)	1491 (32.58)	
BMI (kg/m^2^)	29.10 ± 4.70	27.44 ± 5.19	<0.001
**Waist circumference (cm)**	101.35 ± 10.52	97.25 ± 12.37	<0.001
**Diabetes mellitus**	1568 (81.84)	348 (18.16)	<0.001
**Duration of diabetes (years)**	9.50 ± 26.97	9.97 ± 8.17	0.773
**Hypertension**	1927 (76.02)	608 (23.98)	<0.001
**Current smoker**	765 (63.54)	439 (36.46)	<0.001

*Independent sample t-test or Chi-square test, as appropriate.

**Table 4:** Univariate and multivariable logistic regression analysis of factors associated with dyslipidemia.

	Crude OR (95%CI)	p-value	Adjusted OR (95%CI)	p-value
**Age (years)**	1.004 (0.99, 1.01)	0.285	-	-
**Gender (male)**	0.58 (0.51, 0.65)	<0.001	0.56 (0.47, 0.68)	<0.001
Level of education*				
Middle or high school	0.89 (0.78, 1.02)	0.102	1.08 (0.92, 1.27)	0.325
Academic degree	0.76 (0.63, 0.91)	0.004	0.98 (0.79, 1.22)	0.855
Marital status (Married)†	0.61 (0.49, 0.76)	<0.001	0.90 (0.68, 1.19)	0.467
BMI (kg/m^2^)	1.08 (1.06, 1.09)	<0.001	0.98 (0.95, 1.008)	0.160
**Waist circumference (cm)**	1.03 (1.02, 1.04)	<0.001	1.03 (1.02, 1.04)	<0.001
**Diabetes mellitus**	2.87 (2.50, 3.29)	<0.001	2.28 (1.96, 2.66)	<0.001
**Duration of diabetes (years)**	0.998 (0.991, 1.006)	0.779	-	-
**Hypertension**	2.23 (1.98, 2.52)	<0.001	1.59 (1.38, 1.83)	<0.001
**Current smoker**	0.71 (0.60, 0.85)	<0.001	1.16 (0.93, 1.45)	0.177

OR: odds ratio, CI: confidence interval, A dashed line indicates that the variable was not a candidate for inclusion in the multivariable model

*primary school or lower considered as the reference category

†the category of single individuals was considered the reference category. In the multivariable model, each of the odds ratios were adjusted for other included variables in the model.

**Fig 1:**
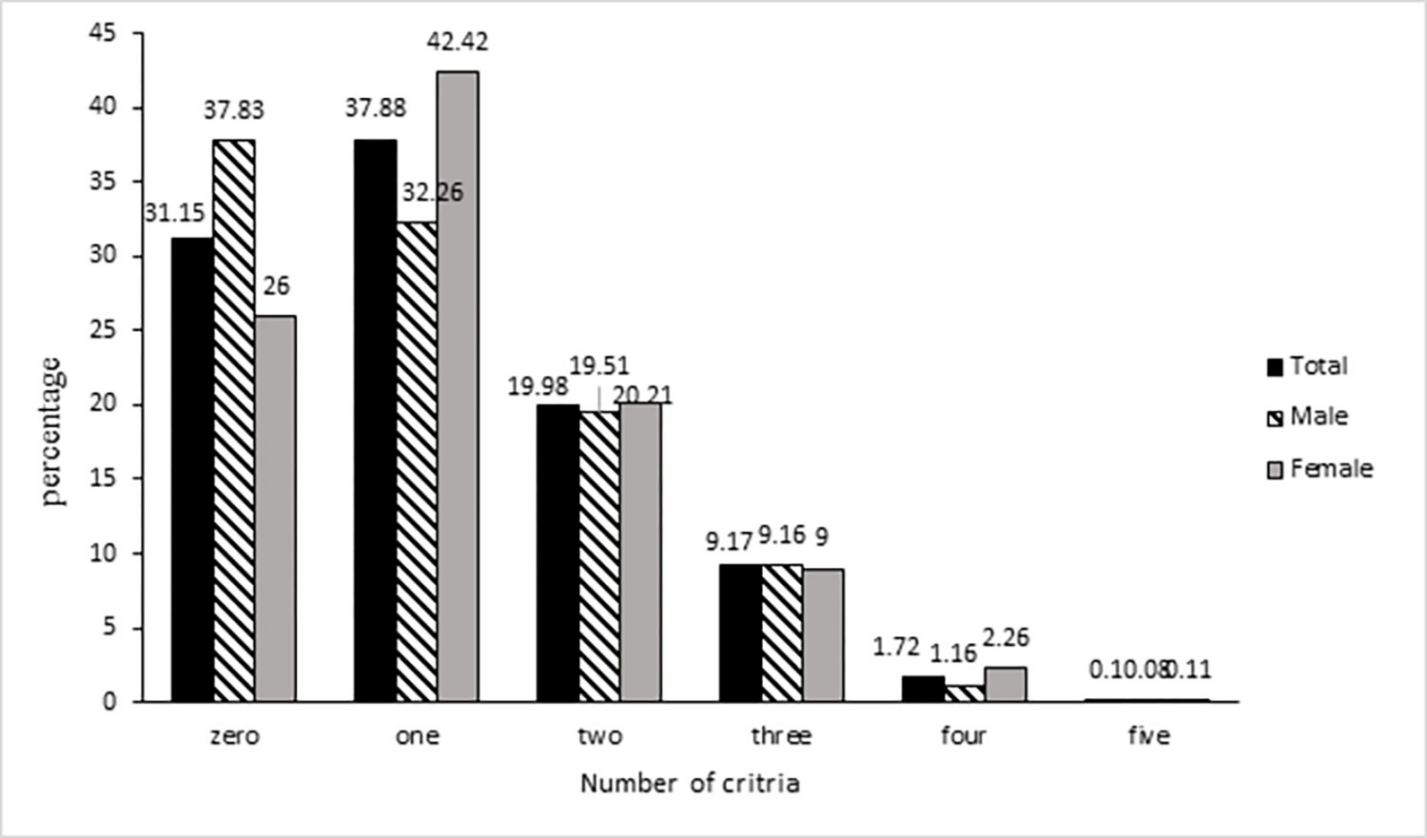
The frequency distribution of number of dyslipidemia components by gender.

Among the participants with dyslipidemia, the majority of males (32.26%) and also females (42.42%) had one component of dyslipidemia (Fig 1).

## Discussion

The purpose of this study was to establish dyslipidemia prevalence and related risk factors in a sample of adults older than 50 years in Iran. We found that more than half of the participants had dyslipidemia. This finding is in line with previous studies including a study conducted among adults aged 65 and older of U.S. population, which have showed a prevalence of 60.3% for dyslipidemia [25]. Further investigation in Chinese adults with a mean age of 60.4±10.0 years has shown that the prevalence of dyslipidemia was 56.1% [26]. Moreover, a study on a group of elderly participants with a mean age of 68.8±3.7 years conducted in Germany, reported that the prevalence of dyslipidemia was 76% [27]. In another study performed among Iranian population, dyslipidemia prevalence was found to be 82.1% and 78.8% in 55–64 and 65–74 years-old individuals, respectively [15]. Moreover, a study conducted among individuals aged over 60 years residing in Bushehr, Iran, dyslipidemia was observed among 87.8% of women and 80.2% of men [14]. A possible explanation for the high prevalence of dyslipidemia in our study could be the high prevalence of its risk factors. So that, our findings indicated a high prevalence of DM, HTN, smoking, and higher mean of BMI and WC in patients with dyslipidemia.

Dyslipidemia is the main predisposing factor of developing cardiovascular disease (CVD) and stroke [28]. CVD is also one of the main causes of morbidity and mortality in old ages [29]. Therefore, timely diagnosis, lifestyle modification, and drug treatment of dyslipidemia can have potential clinical benefits in this population. By lowering blood lipid levels, the risk of CVD and stroke also decreases. The observed high prevalence of dyslipidemia in this study can remind us the importance of considering three essential factors (i.e., screening, lifestyle modification, and treatment) in the elderly population of Iran.

In all age groups, TC, LDL-C, and TG increase with age [30, 31]. However, HDL-C may not decrease with increasing age [31, 32]. Among the different criteria of dyslipidemia, hypercholesterolemia is the most prevalent of them [33]. The frequency of individuals with high TC in our study was 9.74% (12.24% in women, 7.06% in men). In a study performed by Lu et al. on a large Chinese population with a mean age of 55.8±9.8 years, the prevalence of high TC, defined as TC ≥240 mg/dL, was 7.1% [34]. Moreover, according to data from the National Health and Nutrition Examination Survey during 2015–2018, the prevalence of high TC, defined as TC greater than or equal to 240 mg/dL, in U.S. population aged over 60 was 11.4% [35]. In the TLGS, in the age group of 55–64 years, mean TC was 221 and 252 mg/dL for male and female participants, respectively [13]. Our finding in terms of prevalence of high TC is consistent with those reported by previous studies. Possible explanations may include using similar criteria to define elevated TC, and lifestyle factors.

High LDL-C, as a main risk factor for ischaemic heart disease [33], was observed among 5.54% of our sample, with a sex difference of 6.50% in women and 4.51% in men. Lu et al. also found that prevalence of high LDL-C was 4.0% [34]. Moreover, in the TLGS, the prevalence of high LDL-C was 23% in all ages, and the mean LDL-C was 145 and 155 mg/dL in the age group of 45–55 and 55–64 years, respectively [13].

In the current study, of all lipid abnormalities, hypertriglyceridemia was more frequent. So that, the prevalence of high TG was 24.66% (26.22% in women, 23.0% in men). The investigation of Lu et al. estimated the prevalence of high TG as 16.9% [34]. In the National Health and Nutrition Examination Survey, the prevalence of hypertriglyceridemia among U.S. people over 60 years was 28.2%, being higher in female participants than in males (30.9% vs. 24.8% in women and men, respectively) [36]. The mean TG values were 173 mg/dL in all age groups of TLGS, and for 45-54- and 55-64-years age groups were 200 and 206 mg/dL, respectively [13]. The reasons for the high prevalence of high TG in our study may include the comorbidity of DM, obesity, and cigarette smoking.

HDL-C is known to protect against developing CVD. Therefore, a low HDL-C is reversely related to the risk of CVD [37]. The protective role of HDL-C may be related to the function of transporting cholesterol from the arterial wall cells. In the current study a high prevalence of low HDL-C was observed, especially in men. So that, low HDL-C was seen among 19.2% of our study participants (10.27% in women, 28.78% in men). Lu et al. indicated that 15.6% of the individuals had Low HDL-C [34]. In addition, the mean HDL-C values were 43 mg/dL in all age groups of TLGS, and for 45-54- and 55-64-years age groups were 43 and 44 mg/dL, respectively [13]. Elevated TG, high prevalence of cigarette smoking in men, and comorbidity of DM can of the reasons for the high prevalence of low HDL-C.

Primary prevention of CVD through reducing blood lipids is an important issue among older adults. So, lipid lowering medications are more possibly to be used in older patients. In our study, 70.02% of women and 59.53% of men were using antihyperlipidemic drugs. Despite the high frequency of using lipid-lowering drugs, a relatively high prevalence of lipid disorder, especially in terms of high TG, was observed. This indicates that there is still a high number of elderly people who need treatment and lifestyle changes. Or it may even indicate the need for changes in the treatment approach.

Among the possible investigated risk factors of dyslipidemia, gender, educational level, marital status, BMI, WC, DM, HTN, and cigarette smoking showed a statistically significant association. However, after adjusting for the effect of covariates in a multivariable regression analysis, only gender, WC, DM, and HTN remain significant associated risk factors of dyslipidemia. According to Bayram et al., dyslipidemia was positively associated with BMI, WC, fasting blood glucose, and blood pressure in Turkish population [38]. Moreover, Li et al. observed that dyslipidemia prevalence was associated with gender, educational status, smoking, BMI, WC, and DM, in Chinese adults [39]. Another study on the Chinese population found an odds ratio of 1.02 (95%CI: 1.01 to 1.03) in regression analysis for diastolic blood pressure related to dyslipidemia [26]. Our study findings are in line with the previous investigations. We observed that the odds of dyslipidemia was significantly lower in men than in women. This could be due to the stimulatory effect of estrogen in women and the inhibitory effect of androgen in men on lipoprotein metabolism [40]. Considering that a statistically significant association was observed between dyslipidemia and modifiable risk factors such as central obesity, DM, and HTN, it seems reasonable to conduct additional studies on the role of intervention on these factors in the Iranian elderly population.

Age is a physiologic factor that have a considerable impact on levels of blood lipid [30, 32]. Similarly, menopause in women often increases LDL-C, and after the age of 50, women often have higher TC levels than men of the same age [32]. Aging itself causes changes in lipid profile through changes in hormones levels. On the other hand, the limitation in physical activity and subsequently occurrence of obesity leads to insulin resistance, gluconeogenesis and increased production of LDL-C [32]. Furthermore, evidence have shown that there is an association between dyslipidemia and HTN [41]. Therefore, the treatment of dyslipidemia has beneficial effects on blood pressure, and some antihypertensive drugs also affect the lipid profile [41]. It should also be considered that lipid disorders are associated with other cardiovascular risk factors. For example, in this study, we observed such an association with central obesity, DM, and HTN. Therefore, in addition to specific treatments for dyslipidemia, attention should also be paid to controlling these risk factors.

Because of the representative sample used in the present study, the results could be generalizable to the target population. Therefore, the results of this study provide evidence-based decision-making for health policymakers. however, because of the cross-sectional nature of this research, the causal associations between the possible risk factors and dyslipidemia cannot be inferred. In addition, our information about marital status, literacy, smoking and medication use was based on questions from the participants. So, considering this issue, it may be better to be cautious about the results of these variables. In addition, although the prevalence of dyslipidemia may be different in subgroups of patients with and without atherosclerotic cardiovascular diseases, it was not possible for us to estimate the prevalence of dyslipidemia in these patients separately. Another limitation is that in this study, all possible factors related to dyslipidemia, such as genetic factors, nutrition, physical activity, etc., were not investigated.

## Conclusions

The findings of this study indicate that dyslipidemia has become a major health concern in Iranian elderly people. More than half of the study population had dyslipidemia. While high TC, high TG, and high LDL-C were more frequent in women; low HDL-C was more frequent among men participants. lifestyle modification along with antihyperlipidemic drugs can improve lipid profile in the elderly. We also observed that more than half of the participants with dyslipidemia had a primary educational level. This can affect the knowledge of following a healthy lifestyle and the need for timely treatment, so health education can be used as a strategy to reach the desired lipid level.
